# Apoptosis induction on human breast cancer T47D cell line by extracts of *Ancorina *sp.

**DOI:** 10.12688/f1000research.17584.2

**Published:** 2019-04-10

**Authors:** Woro Anindito Sri Tunjung, Puspa Restu Sayekti

**Affiliations:** 1 Faculty of Biology, Universitas Gadjah Mada, Yogyakarta, 55281, Indonesia

**Keywords:** Ancorina sp., cytotoxicity, apoptosis, caspase-3, breast cancer

## Abstract

**Background: **Breast cancer is the second leading cause of death in women. Alternative medicine with high efficacy is needed for breast cancer treatments, for example induction of apoptosis using natural products. It has been found that many natural apoptosis-inducing compounds are isolated from marine sponge. The objective of this study is to analyze the ability of extracts of the sponge *Ancorina* sp. to induce apoptosis on human breast cancer T47D cell line and find out its mechanism.

**Methods: **T47D cells were treated with crude extracts of methanol, dichloromethane:methanol (1:1) and dichloromethane *Ancorina* sp. for 24 h, and doxorubicin was used as a positive control. Methods used for this study were MTT assay to examine cell viability and determine IC _50_ of the three extracts, while the percentage of apoptosis and caspase-3 were investigated by flow cytometry.

**Results: **IC _50_ values of methanol, dichloromethane:methanol (1:1), and dichloromethane extract were 84.25, 121.45, and 99.85μg/mL respectively. The percentages of apoptotic cells after treatment with methanol, dichloromethane:methanol (1:1), and dichloromethane extracts were 88.68, 27.54 and 53.63% respectively, whereas the percentage of caspase-3 was 77.87, 12.66 and 12.97%, respectively.

**Conclusions: **These results revealed that all extracts of *Ancorina *sp. have strong or moderate cytotoxicity and have the ability to induce apoptosis on T47D human breast cancer cell line. However, methanol crude extract has high efficacy to induce apoptosis through caspase-3 activation compared to the other extracts. Hence methanol extract warrants further investigation as a natural medicine for human breast cancer.

## Introduction

Breast cancer is the second leading cause of death in women after cervical cancer. In 2016 breast cancer cases have occurred in 40 per 100,000 women in Indonesia ^1^. Medical treatment for breast cancer is currently widely applied ^2^. However, medical treatment can cause side effects, namely the death of healthy cells surrounding cancer cells ^3^. Alternative methods of breast cancer treatment with reduced side effects are needed, such as treatments using natural anticancer agents ^3^.

There are many cancer treatment methods such as anti-angiogenesis therapy ^4^, cell cycle inhibitors ^5^, and photodynamic therapy ^6^. Induction of apoptosis is the most common approach in cancer therapy because apoptosis has specific abilities to kill certain cells ^7^. One characteristic of cancer cell is loss of ability for apoptosis ^8^. The ability of apoptosis to kill abnormal cells can prevent the occurrence of cancer growth ^9^. Induction of apoptosis occurs through three apoptotic-signaling pathways:extrinsic, intrinsic and perforin/granzyme pathways. Apoptosis path activation is marked by the activation of caspases. Caspase is found in normal cells as an inactive zymogen (procaspase). Active caspase activates other caspases, forming the ‘caspase cascade’. Activation of caspase 8 and 9 will cause activation of caspase-3 as a downstream effector, which induces apoptosis ^10^.

Previous studies found many natural apoptosis-inducing compounds isolated from marine sponge that can be developed as natural medicine ^11^. Fraction of *Negombata magnifica* sponge is able to induce apoptosis in hepatocellular carcinoma ^12^. Sponge extract of *Haliclona* sp. able to increase the percentage of apoptosis and significantly increase the expression of apoptotic gene p53, p21, caspase-8, and caspase-3 in A549 lung cancer cells ^13^.

Natural anticancer agents are usually extracted by a particular solvent. Different solvents cause different effects on the disease. Some previous researchers have isolated sponge bioactive compounds using both polar and non-polar solvents. For example, cytotoxic compounds have been successfully isolated from sponge *Dactylospongia elegans* and *Pachychalina alcaloidifera* using methanol ^14, 15^. Organic compounds have been successfully isolated from the sponge *Condrosia reniformes, Tethya rubra, Tethya ignis, Mycale angulosa* and *Dysidea avara* as a drug therapy for Chagas disease using acetone solvents ^16^. Terpenoids have been successfully isolated from sponge *Iricina* sp. and *Spongia* sp. using ethanol solvent ^17^. Anticancer compounds have been successfully isolated from *Petrosia* sp., *Jaspis* sp. and heterogeneous *Pericharax* using dichloromethane:methanol (1:1) ^18^. Some studies also mention that sponge bioactive compounds, antiviral, antimicrobial, antifungal, and anticancer compounds, have been successfully isolated with methanol ^19– 21^, ethanol ^22^, dichloromethane and combination of dichloromethane:methanol (1:1) ^23– 26^.

The objective of this study is to determine the cytotoxicity of *Ancorina* sp. extract in breast cancer T47D cells and measure extract-induced apoptosis through activation of caspase-3. In this study we use three solvents: methanol (polar), dichloromethane (non-polar) and mixture of both solvents to determine the most effective solvent. Furthermore this study used T47D cells as a model for breast cancer cells because T47D cells are able to express caspase-3, which is an effector of apoptotic induction ^27^.

## Methods

### Sample preparation and determination

*Ancorina* sp. were collected from Wedi Ombo Beach, Gunungkidul, Yogyakarta, Indonesia. Samples were washed to remove debris and residual salt. Samples were transferred to the laboratory in methanol, dichloromethane and dichloromethane:methanol (1:1) under cool condition.

### Extraction

Fresh samples were crushed in a blender in methanol, dichloromethane and dichloromethane methanol (1:1) then macerated for 24 hours. The samples were filtered using whatman no 1 (Sigma) and the residue was re-extracted for two times. The total filtrate was then naturally air drying in room temperature to obtain crude extract paste.

### Cell line culture

We used T47D cells obtained from Integrated Laboratory of Research and Testing, Universitas Gadjah Mada (LPPT UGM).

The cells were cultured in RPMI 1640 medium supplemented with 10% FBS, 2% penicillin streptomycin and 0.5% Fungizone. Cells were harvested after reaching 80% confluence using 0.25% Trypsin-EDTA. Cells were cultured in 96-well microplates (1 × 10 ^3 ^cells/well) in 100 μL RPMI and incubated at 37°C with 5% CO _2_ overnight.

Doxorubicin at 5 µg/mL was used as the positive control whereas T47D cells cultured in medium was used as the negative control and cells cultured in 0.5% DMSO in medium was used as the solvent blank.

### Cytotoxicity assay

Cytotoxicity was assessed using the MTT assay. After the cells were incubated for 24 h with the serial dilution 15.68, 31.25, 62.50, 125 to 250 µg/mL of extract, 0.5% MTT solution was added and the cells were incubated for 4 h followed by addition of stopper reagent (10% SDS in 0.1 N HCl). Each treatment was subjected with 3 replication. Those serial concentration is based on preliminary experiments. The optical density (OD) was measured at 550 nm using Microplate Reader BIO-RAD 680XR. The percentage of cell viability was obtained by this formula:

$$\frac{\text{Absorbance}\;\text{of}\;\text{treatment}}{\text{Asorbance}\;\text{of}\;\text{control}\;\text{cell}}\;\times \;100\%$$

Inhibitory Concentration 50% (IC _50_) of Ancorina sp. was then determined by probit analysis using value among cell viability and log concentration of extracts. IC _50_ of each extract is used for FACS experiment.

### Apoptosis and caspase-3 assay

Briefly, T47D cells were seeded in 6-well microplates in 3×10 ^3^μL RPMI. In total, 1×10 ^6^ cells were treated by IC _50_ concentrations of three extracts or doxorubicin for 24 h. Cells were stained by Annexin V-PI Biolegend ** for apoptosis test and by BD Cytofix / Cytoperm™ for caspase-3 activation test. The sample was measured using flow cytometer BD FACSCalibur™. Flowcytometry output by BD FACSCalibur™ was shown in four quadrants. The first quadrant contains normal living cells population that respond negatively to Annexin V-FITC and propidium iodide (PI). Second quadrant contains early apoptotic cells populations that respond positively to Annexin V-FITC. Third quadrant contains the late apoptotic cells population which responds positively to Annexin V-FITC and Propidium Iodide (PI). Whereas, the fourth quadrant contains a population of necrotic cells that respond negatively to Annexin V-FITC and respond positively to PI ^28^. On the other hand, in the caspase-3 test the black area indicated control cells while R1 showed caspase-3 activated cells.

### Data analysis

The IC _50_ value was determined by Probit analysis. IC50 value and percentage of apoptosis are further analyzed by one-way ANOVA and Tukey’s test at 5% significance level using IBM SPSS Statistic 23.0 program. P < 0.05 indicated statistical significance.

## Results

### Cytotoxicity

**Figure 1.**
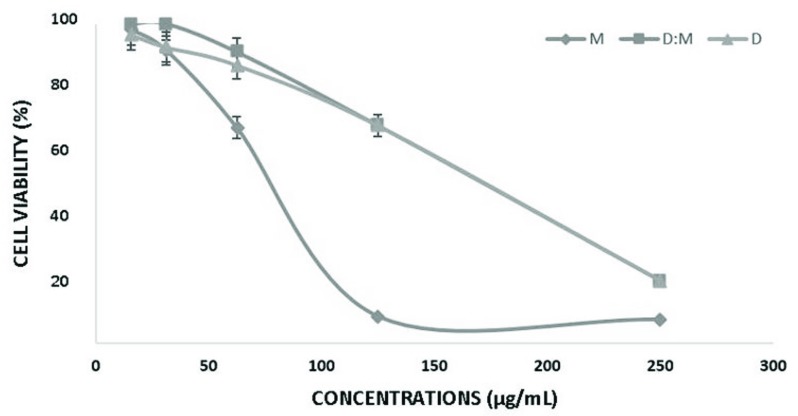
Breast cancer T47D cell viability after treatment with extracts of *Ancorina* sp. Methanol (M), Dichloromethane:Methanol (D:M) and Dichloromethane (D). Error bar shows standard deviation.

The cell viability of T47D cells after methanol, dichloromethane and dichloromethane: methanol (1:1) extracts treatment are presented in Figure 1. The concentration of extracts reduced the viability of investigated cells by 50% (IC _50_), which has been reported in Table 1.

**Table 1.** IC _50_ values of *Ancorina* sp. extracts.

Treatments	IC _50_ value (µg/mL)
Methanol	84.25 ^a^ ± 9.52
Dichloromethane:methanol (1:1)	121.45 ^b^ ± 10.11
Dichloromethane	99.85 ^ab^ ± 11.79

Note: different letter showed the significant difference at the 0.05 level

All *Ancorina* sp. extracts inhibited the proliferation of cancer cells in a dose dependent manner. The higher concentration of extract caused the lower percentage of T47D cell viability. All extracts were cytotoxic to T47D cells. IC _50_ value of methanol was significantly different to dichloromethane:methanol but wasn’t significantly different to dichloromethane.

### Apoptosis and caspase-3 activation assay

We analyzed cell death qualitatively by examining cell morphological change and quantitatively by flow cytometry using Annexin-V after 24 h incubation of extracts.

The DMSO treated cell and control showed living cells withnormal morphology. T47D cells in these groups form tightly cohesive mass structures displaying robust cell-cell adhesions. However, after doxorubicin and extract treatment most cells undergo death. The cells became shrunken and showed signs of detachment from the surface of the wells, which denoted cell death

**Figure 2.**
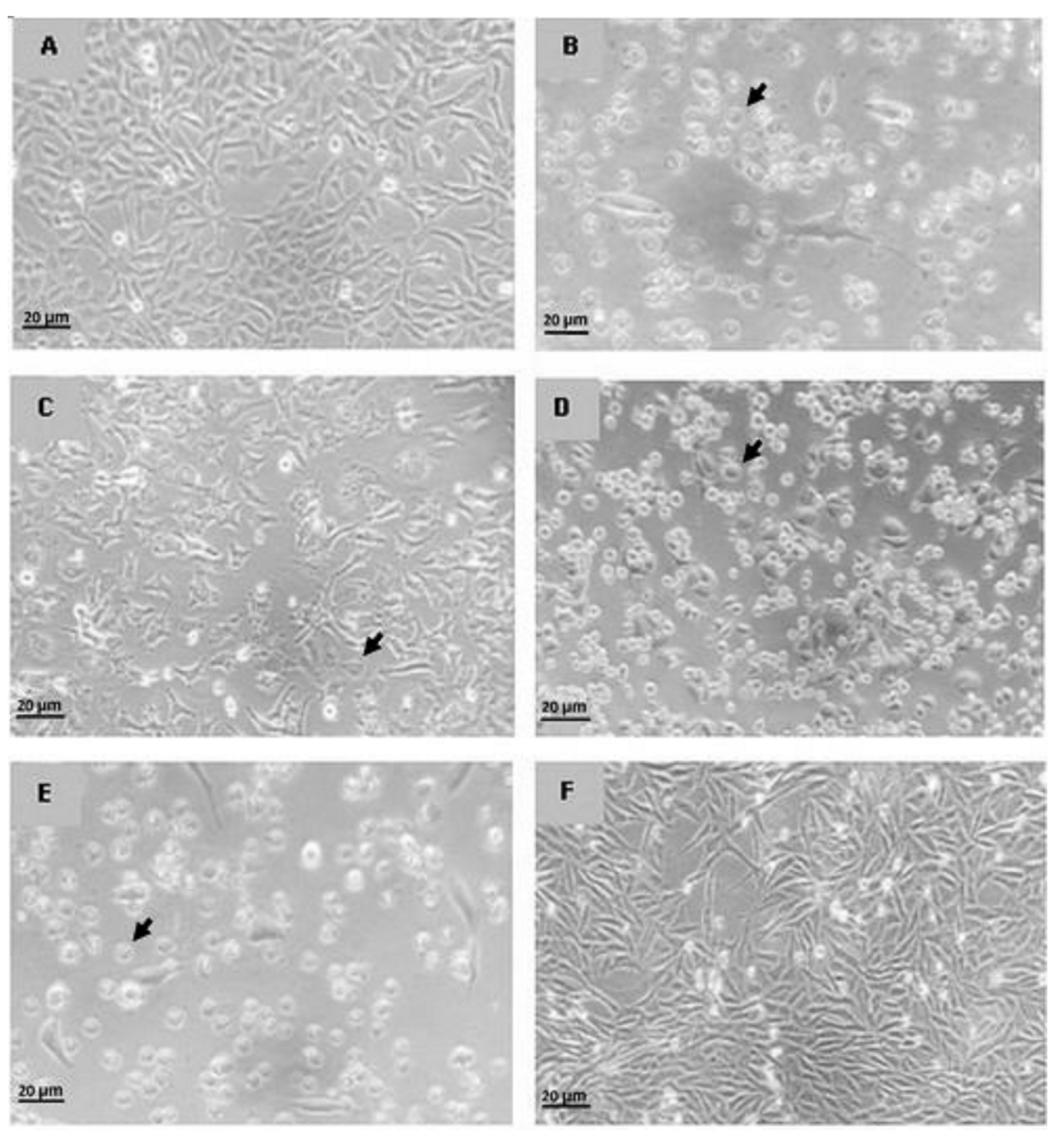
Cell morphology of breast cancer T47D cells after treatment with extracts of *Ancorina* sp. Control ( **A**), Methanol ( **B**), Dichloromethane:Methanol ( **C**), Dichloromethane ( **D**), Doxorubicin ( **E**) and DMSO ( **F**). Observation of cell morphology was performed using inverted microscope Axio Vert.A1 Zeiss with a magnification of 40x. Arrow shows dead cells.

Cell morphology after treatment can be seen in Figure 2. Morphology of T47D cell showed that methanol extract caused most cells population to undergo death (approximately more than 70%), while dichloromethane extract resulted in almost half cell population deaths. The combination of methanol and dichloromethane (1:1) extract causes fewer cell deaths (<50%). This data supports the cytotoxicity assay that the *Ancorina* sp. extracts successfully induced cell death

**Figure 3.**
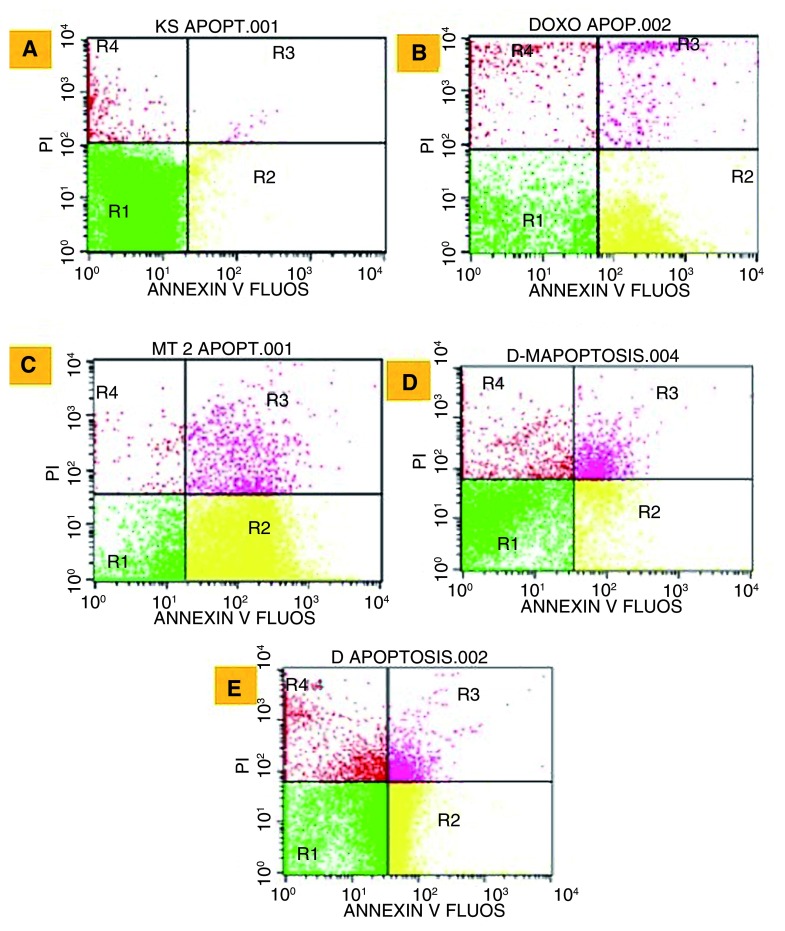
Detection of apoptosis markers of T47D cell. Cell control ( **A**), doxorubicin ( **B**), methanol ( **C**), dichloromethane:methanol (1:1) ( **D**), and dichloromethane ( **E**). R1 (normal cells), R2 (early apoptosis cells), R3 (late apoptotic cells), and R4 (necrosis cells).

Detection of apoptosis marker after treatment by extracts can be seen in Figure 3.

All *Ancorina* sp. extracts increase the percentage of apoptotic cells compared to control cells ( Figure 3). The highest percentage of apoptosis was obtained in the methanol group (88.68%), which was even higher than doxorubicin as a positive control (75.74%) ( Table 2).

**Table 2.** Percentage of T47D cell population after treatment of crude extracts of *Ancorina* sp.

Treatment	Applied concentration (IC _50_ value)	Percentage of cell (%)		
		Normal	Early + Late apoptosis	Necrosis
Methanol	84.25	6.94 ± 0.21	88.68 ^a^ ± 0.47	4.38 ± 0.25
Dichlorometane : Methanol (1 :1)	121.45	65.51 ± 2.79	27.54 ^b^ ± 0.93	7.37 ± 1.65
Dichlorometane	99.85	39.29 ± 1.60	53.63 ^c^ ± 1.42	7.60 ± 0.91
Doxorubicin	5	20.65 ± 2.09	75.74 ^d^ ± 1.58	3.67 ± 0.44
Negative control	-	92.93 ± 0.01	1.84 ^e^ ± 0.15	5.34 ± 0.21

Note: Value after ± shows standard deviation of two replications. Different letter showed the significant difference at the 0.05 level. Statistical analysis is focused to percentage of apoptosis among group.

The three extracts showed the same pattern with doxorubicin, i.e. a high percentage of apoptotic cell while the percentage of necrotic cells is low ( Table 2).

**Figure 4.**
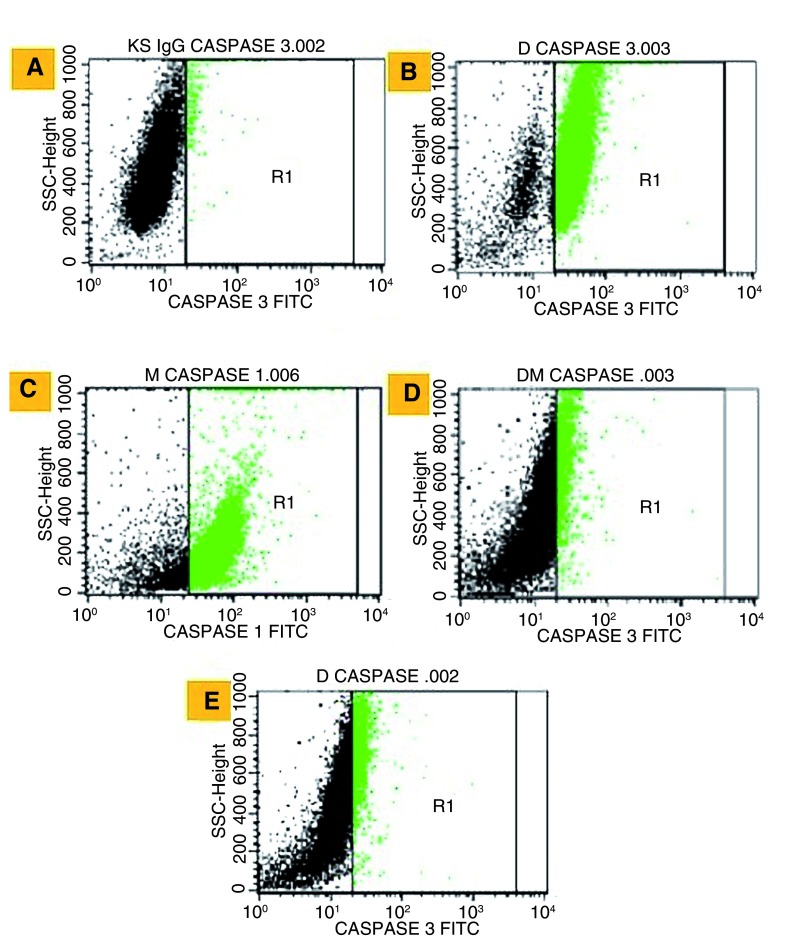
Detection of caspase-3 activation in breast cancer T47D cells after treatment with extracts of *Ancorina* sp. Negative control ( **A**), Doxorubicin ( **B**), Methanol ( **C**), Dichloromethane:Methanol ( **D**) and Dichloromethane ( **E**). Black area indicated control cells while R1 showed caspase-3 activated cells.

**Figure 5.**
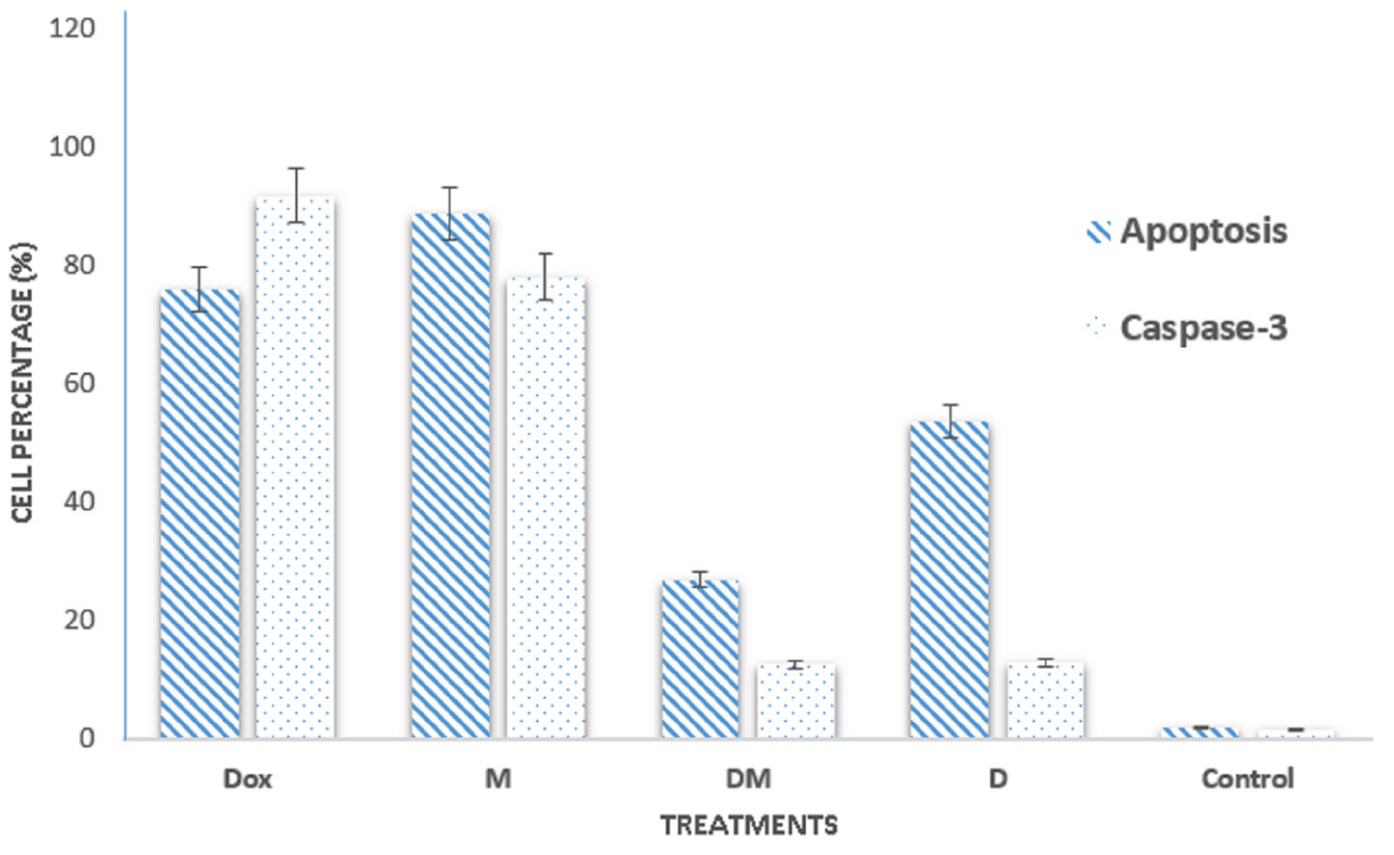
Correlation between percentage of apoptosis and caspase 3 activation in breast cancer T47D cells after treatment with extracts of *Ancorina* sp. Doxorubicin (Dox), Methanol (M), Dichloromethane:Methanol (1:1) (D:M), Dichloromethane (D), and negative control (control). Value after ± and error bar show standard deviation of two replications.

We further investigated the apoptotic mechanism by examining the percentage of caspase-3. Detection of caspase-3 can be seen in Figure 4, while percentage of caspase-3 activation and correlation between percentage of apoptosis and caspase-3 activation can be seen in Table 3 and Figure 5, respectively.

**Table 3.** Percentage of caspase-3 activation after treatment by crude extracts of *Ancorina sp.*

Treatments	Applied concentrations IC _50_(µg/mL)	Caspase-3 (%)
Methanol	84.25	77.87 ± 5.81
Dichlorometane : Methanol (1:1)	121.45	12.66 ± 3.30
Dichlorometane	99.85	12.97 ± 2.11
Doxorubicin	5	91,53 ± 4.09
Negative control	-	1.54± 0.00

The highest percentage of caspase-3 was detected with methanol extract, which almost equaled doxorubicin, while the value of the other extracts was lower ( Table 3).

The three extracts have a positive correlation between percentage of apoptosis and caspase-3. Although dichloromethane showed lower percentage of apoptosis and caspase-3, but they still have strong cytotoxicity (99.85 µg/mL), which shows potency as natural anticancer agents.

## Discussion

Sponges are highly diverse in Indonesia. In particular, encrusting sponges abundantly live in Gunung Kidul, Yogyakarta. Marine sponges produce some secondary metabolites, which can be used as antiviral ^22^, antimicrobial ^22, 23^, antifungal ^22^, and anticancer drugs ^17, 24, 29^. The cell adhesion and immune system in sponge allow the different forms of the body plan ^30^. When encrusting sponges grow together, sponges can survive by producing chemicals to kill fast dividing cells from the neighboring sponges. This ability of the chemicals can be used for chemotherapy since the basis of chemotherapy treatments is to disturb cancer cell growth ^31^.

Sponge *Ancorina* sp. is a member of family Ancorinidae, which contains bioactive compounds such as ancorinoside BD, penazetidine A ( *Penares sollasi*), ecionines A & B ( *Ecionemia* sp.) and Iso malabaricane triterpenes ( *Rhabdastrella globostellata*) ^32^. Ancorinoside is a MT1-matrix metalloproteinase inhibitor in the development and metastasis of tumor cells ^33^, whereas Penazetidine A strongly inhibits PKC-β1 activity in tumor cells with IC _50_ value 0.3 μg / mL ^34^.

Ecionines A (biemnadin) and B (meridine) are anticancer compounds for many cancer cells, including bladder cancer cells ^33^. Further, Iso malabaricane triterpenes were also found to be anticancer after testing on three types of cancer cells, namely L5178Y (mouse lymphoma), HeLa (human cervical carcinoma), and PC-12 (pheochromocytoma in mice) ^35^. *Ancorina* sp. is a source of bioactive compounds such as ancorinoside A Mg salt, ancorinolates AC, bis-ancorinolate B, ancorinazole, indolo [3,2-a] carbazole, and (+) - 7-bromotrypargine ^36– 38^. These previous data show the high potency of Ancorinidae to be used as natural anticancer agents; hence this study is focused on the potency of *Ancorina* sp. as an anticancer agent and its mechanism, which is possibly through apoptosis induction.

Cytotoxicity is categorized into three levels by IC _50_ extract values. Very strong cytotoxicity has IC _50_ less than 10 μg / mL, strong cytotoxicity has IC _50_ values between 10 –100 μg/mL, and moderate cytotoxicity has IC _50_ values between 100 – 500 μg/mL ^39^. According to these ranges, IC _50_ of the methanol and dichloromethane extracts in the present study had strong cytotoxic ability, while dichloromethane:methanol (1:1) extract belonged to moderate cytotoxicity. *Ancorina* sp. extracts have greater value of IC _50_ compared with the study ^34^, which mentioned penazetidine A, a bioactive compound of marine sponge and highly inhibits PKC-β1 activity in tumor cells with lower IC _50_ of 0.3 μg/mL. This difference is due to the non-fractionated extract of our sponge, so that unsorted bioactive compounds possibly affect the cytotoxicity ability of extracts ^34^.

Bioactive compounds from natural products depend on solvents. Based on the polarity of solvents, in the present study, *Ancorina* sp. extracts with polar solvent (methanol) lead to a higher apoptosis than non-polar (dichloromethane) or combination. These results are supported by a previous study that showed some compounds of Ancorinidae, such as ancorinoside BD, penazetidine A, echionines A and B and isomalabaricane triterpenes, are polar compounds that have anti-tumor and anti-cancer characteristics ^32^. Interestingly, some studies in sponge also show same phenomenon such as cytotoxic compounds from sponge *Dactylospongia elegans* and *Pachychalina alcaloidifera* has been isolated using polar solvent methanol ^14, 15^. Terpenoids from sponge *Iricina* sp. and *Spongia* sp. have been isolated using polar solvent ethanol ^14, 15, 17^. Bioactive compounds of sponge, both antiviral, antimicrobial, antifungal, and anticancer compounds have been successfully isolated by polar solvent as methanol ^19– 21^ and ethanol ^22^. Considering all extracts in this study have low necrosis values ( Table 2), they are safe to be used as medicine. Therefore, further studies are needed to find out the specific compounds of *Ancorina* sp. extracts.

Apoptosis can be triggered by extrinsic stimulation through death receptors on cell surfaces, such as TNFα (Tumor Necrosis Factor-α), Fas receptor (CD95 / APO1) and TRAIL (TNF related to ligand-inducing apoptosis) or by intrinsic stimulation through mitochondrial signaling pathways. In these two main pathways, activation of cysteine aspartyl proteases or caspase can produce mitochondrial permeabilization membrane, chromatin condensation and DNA fragmentation. These events stimulate the cells that are undergo apoptosis and lead to a distinctive cell morphology, such as the appearance of pyknosis, chromatin condensation, nucleus fragmentation, and apoptotic body formation, but organelles are still intact ^40^. This can be seen in the present study in Figure 2.

Apoptotic pathways commonly occur by the activation of caspase-3, which is the effector of intrinsic, extrinsic and perforin pathways ^41^. Caspase-3 is a key protease that is activated during the early stages of apoptosis. Caspase-3 is proteolytically active, cuts and activates other caspases, as well as relevant targets such as targets in the cytoplasm (D4-GDI and Bcl-23) and nucleus (poly (ADP-ribose) polymerase; PARP1) ^42^.

In the present study, the highest percentage of caspase-3 was detected in methanol extract, which almost equal to doxorubicin, while the other extracts was lower ( Table 3). Doxorubicin as a commercial drug in chemotherapy revealed a high percentage of apoptotic cells and caspase-3 activation. Among *Ancorina* sp. treatment groups, methanolic extract showed the highest percentage of both apoptosis and caspase-3. Interestingly, the methanolic extract showed a higher percentage than doxorubicin, and revealed its great potency to be used as a cancer medicine ( Table 3).

The three extracts in this study have a positive trend between percentage of apoptosis and caspase-3 activation. Although dichloromethane showed a lower percentage of apoptosis and caspase-3, they had strong cytotoxicity (99.85 µg/mL) which shows potential as natural anticancer agents. It is possible that anticancer mechanism of dichloromethane and mixture of dichloromethane and methanol (1:1) excludes caspase-3 activation as effector caspases. Another pathway, such as caspase-6 or 7, can also induce apoptosis in T47D breast cancer cells ^27^. More investigation is needed to elucidate the anticancer mechanism of these extracts.

## Conclusions

All extracts of *Ancorina* sp. have strong or moderate cytotoxicity and have the ability to induce apoptosis in T47D human breast cancer cell line.

## Data availability

### Underlying data

Open Science Framework: Apoptosis induction on human breast cancer T47D cell line by extracts of *Ancorina* sp., https://doi.org/10.17605/OSF.IO/AEJ96 ^43^

Data are available under the terms of the Creative Commons Zero “No rights reserved” data waiver (CC0 1.0 Public domain dedication).
