# Supplementary material for: Apoptosis induction on human breast cancer T47D cell line by extracts of Ancorina sp
Source: F1000Res. 2019 Apr 10;8:168. Originally published 2019 Feb 7. [Version 2] doi: 10.12688/f1000research.17584.2 (PMC6468741; doi:10.12688/f1000research.17584.2)
Supplement: Peer review history

# Peer review: Reviewer response for version 2

**DOI:** 10.5256/f1000research.20632.r47034

**Published:** 2019-04-16

**Authors:** Sutiman Bambang Sumitro

**Affiliations:** 1 Department of Biology, Faculty of Sciences, Brawijaya University, Malang, Indonesia

All of my comments are already sufficiently responded to.

I have read this submission. I believe that I have an appropriate level of expertise to confirm that it is of an acceptable scientific standard.

# Peer review: Reviewer response for version 2

**DOI:** 10.5256/f1000research.20632.r47035

**Published:** 2019-04-12

**Authors:** R. Luke Daniels

**Affiliations:** 1 Department of Biology, The College of Idaho, Caldwell, ID, USA

The authors have addressed the comments made regarding previous drafts, and the methods and figures are now substantially improved.

I have read this submission. I believe that I have an appropriate level of expertise to confirm that it is of an acceptable scientific standard.

# Peer review: Reviewer response for version 1

**DOI:** 10.5256/f1000research.19228.r44188

**Published:** 2019-03-21

**Authors:** R. Luke Daniels

**Affiliations:** 1 Department of Biology, The College of Idaho, Caldwell, ID, USA

This paper describes the cytotoxic effects of extracts from a marine sponge. The authors provide evidence that extracts were cytotoxic, and that methanol-extracts were the most cytotoxic (as measured by MTT assay). Additional studies showed that the mechanism of cell death is likely through apoptosis (as measured by examining Annexin V/PI staining and caspase 3 activity via flow cytometry). These are novel results that contribute to the search for anti-cancer agents, and it is this reviewer’s opinion that indexing is warranted. There are a few minor modifications that I would suggest making before finalizing this manuscript.

**Methods:**

 It would be helpful to include additional details about the methods, such as:

1. specific extraction methods.
2. the method in which the IC50 was calculated.

**Figure 1:**

 This reviewer agrees that the MTT data supports the idea that these various extracts are cytotoxic. In Figure 1, it seems that the y-axis is the percentage of cells that are healthy relative to controls (?), but the units are not given. It would be helpful in Figure 1 to:

1. give the units for the y-axis.
2. provide some explanation of the dose that is given in each treatment (currently given in the methods).
3. know the number of trials this experiment represents.
4. What is the n, what do the error bars represent? (Standard error? Standard deviation?)

It might also be beneficial to do an ANOVA to better understand whether differences among these experimental treatments might be statistically significant (in other words, are the dose-dependent changes in cell viabilities statistically different from each other?). I do not consider this to be essential for indexing.

**Figure 2:**

 This reviewer agrees that these treatment compounds seem to negatively impact cells (based on their morphology). It would be beneficial to:

1. describe the differences that were observed for each treatment condition, as there seems to be some variation (C vs. E for example).

**Figure 3 & 4:**

 It appears that the flow cytometry experiments were repeated a number of times, as implied by the variability that is reported in Tables 2 & 3 (+/- values). It would be helpful to:

1. give the number of times the experiment repeated.
2. state what this variability represents (standard error? Standard deviation?).

I have read this submission. I believe that I have an appropriate level of expertise to confirm that it is of an acceptable scientific standard, however I have significant reservations, as outlined above.

# Peer review: Reviewer response for version 1

**DOI:** 10.5256/f1000research.19228.r44186

**Published:** 2019-02-26

**Authors:** Sutiman Bambang Sumitro

**Affiliations:** 1 Department of Biology, Faculty of Sciences, Brawijaya University, Malang, Indonesia

**Page 3:**

        

**Methods: Extraction:**

Statement:

 “Fresh samples were crushed in a blender in methanol, dichloromethane and dichloromethane methanol (1:1) then macerated for 24 hours. The samples were filtered and the residue then was re-extracted for two times. The total filtrate was then evaporated to obtain crude extract paste.”

Questions:

1. What is used to filter the extract?
2. What is used to evaporate? What degree of temperature is used to evaporate?
3. What is the type of the tool?

 

Statement:

 “The cells were cultured in RPMI 1640 medium supplemented with 10% FBS, 2% penicillin streptomycin and 0.5% Fungi zone. Cells were harvested after reaching 80% confluence using

 0.25% Trypsin-EDTA. Cells were cultured in 96-well micro plates (1 × 103 cells/well) in 100 μL RPMI and incubated at 37°C with 5% CO2 overnight”.

Suggestion:

 It's better to explain what DMSO, MTT, etc., stand for.

**Page 4:**

**Results** **:**

**Figure 1. Breast cancer T47D cell viability after treatment with extracts of *Ancorina *sp. **Methanol (M), Dichloromethane: Methanol (D:M)

 and Dichloromethane (D).

Questions:

1. What is the unit of the cell viability?
2. The log concentration (?).
3. Add the scaling line next to the numbers.

  

**Page 5:**

**Table 1. IC50 values of *Ancorina *sp. extracts.**

**Treatments IC50 value (μg/mL)**

 Methanol                                             84.25a ± 9.52

 Dichloromethane:methanol (1:1)        121.45 b ± 10.11

 Dichloromethane                                 99.85ab ± 11.79

Note: different letters showed the significant difference at the 0.05 levels.

Questions:

1. How is the IC _50_ calculated?
2. Why do dichloromethane (non-polar solvent) and methanol indicate moderate cytotoxicity, while on the other hand, methanol solvent (polar solvent) indicates the highest cytotoxicity?

 

**Figure 2. Cell morphology of breast cancer T47D cells after treatment with extracts of *Ancorina *sp. **Control ( **A**), Methanol ( **B**), Dichloromethane:Methanol ( **C**), Dichloromethane ( **D**), Doxorubicin ( **E**) and DMSO ( **F**). Arrow shows dead cells.

Questions:

1. What are the morphological differences among the treatments? This needs to be explained. Is it only to show dead and living cells? If this is the case one figure is enough.
2. The figures were taken using what kind of microscope?
3. Add the microscopic scale on the figure.

 

**Page 7:**

**Figure 4. Detection of caspase-3 activation in breast cancer T47D cells after treatment with extracts of *Ancorina *sp. **Negative control ( **A**), Doxorubicin ( **B**), Methanol ( **C**), Dichloromethane: Methanol ( **D**) and Dichloromethane.

Question: What is R1?

**Page 8**:

**Figure 5. Correlation between percentage of apoptosis and caspase 3 activation in breast cancer T47D cells after treatment with extracts of *Ancorina *sp. **Doxorubicin (Dox), Methanol (M), Dichloromethane: Methanol (1:1) (D:M), Dichloromethane (D), and negative control (control).

Suggestion: The figure is not clear. Add the scaling line next to the numbers.

**Page 8:**

*Ancorina *sp. is a source of bioactive compounds such as ancorinoside A Mg salt,

 ancorinolates AC, bis-ancorinolate B, ancorinazole, indolo [3,2-a] carbazole, and (+) - 7-bromotryparg.

Suggestion: Mention the characteristics of these compounds e.g. the level of their polarity.

I have read this submission. I believe that I have an appropriate level of expertise to confirm that it is of an acceptable scientific standard, however I have significant reservations, as outlined above.
